# Idalopirdine – a small molecule antagonist of 5-HT_6_ with therapeutic potential against obesity

**DOI:** 10.1007/s11011-015-9736-3

**Published:** 2015-09-29

**Authors:** Magdalena Dudek, Monika Marcinkowska, Adam Bucki, Adrian Olczyk, Marcin Kołaczkowski

**Affiliations:** Department of Pharmacodynamics, Jagiellonian University, Collegium Medicum, 9 Medyczna Street, PL 30-688 Kraków, Poland; Chair of Pharmaceutical Chemistry, Faculty of Pharmacy, Jagiellonian University, Collegium Medicum, Medyczna 9, 30-688 Kraków, Poland; Institute of Automatic Control, Silesian University of Technology, Gliwice, Poland

**Keywords:** LuAE58054, Idalopirdine, 5-HT6 receptor antagonist, Obesity, Rats with diet-induced obesity, Anorectic activity

## Abstract

5HT_6_ receptor antagonists offer the potential for safe and effective drugs against obesity, because they can reduce weight without causing serious side effects in the cardiovascular system. Also, their anorexic effect is associated with reduced food intake via an enhancement of satiety. In the present study we investigated the anorexic effect of idalopirdine (LuAE58054) in a model of obesity induced by high-fat diet. To induce obesity in rats, the animals were treated with feed with a fat content of 40 %. Body weight was controlled and the amount of food and water consumed was determined. The influence of the test compound on the lipid profile and glucose level was measured, as well as locomotor activity in home cages on the 20th day of the treatment. LuAE58054, at 5 mg kg^−1^/day i.p., was significantly anorectic in this model of obesity. Animals treated with LuAE58054 weighed 8 and 9.2 % less than the control obese animals on the 12th and 21st days, respectively. It significantly reduced food intake and the amount of peritoneal fat in animals, and reduced the level of triglycerides in plasma. LuAE58054 did not have a statistically significant effect on the spontaneous activity of diet-induced obese rats. The present study clearly demonstrates the effectiveness of LuAE58054 in reducing body weight. This compound is in phase III of clinical trials for the treatment of cognitive deficits associated with Alzheimer’s disease and schizophrenia. It is a 5HT_6_ receptor antagonist and is, therefore, free of those unacceptable side effects that preclude chronic use of anti-obesity drugs with other mechanisms of action. The search for an effective and safe anti-obesity drug is essential for an increasingly obese population; therefore, the anorectic action of LuAE58054 is important and there is a need for more research in this direction.

## Introduction

Obesity is a serious problem in many countries. The World Health Organization estimates that it affects between 30 and 80 % of adults and up to 30 % of children, and effective treatment of this disease is one of the major health problems of the 21st century. The World Health Organization has recognized obesity as an epidemic of the 21st century (Zięba 2007).

5-Hydroxytryptamine-6 (5-HT_6_) receptor is a promising molecular target. There is an impressive increase in the number of publications and patents in the 5-HT_6_ receptor field, demonstrating the high level of interest in research in this area over recent years (Jastrzębska-Więsek et al. 2013; Heal et al. 2008). Since it has been discovered that 5-HT_6_ receptor knock-out mice are resistant to high-fat diet-induced obesity and since a decrease in food intake and body weight in 5-HT_6_ receptor knock-out mice has been observed (Caldirola 2003; Frassetto et al. 2008), researchers have begun to investigate the 5HT_6_ receptor ligands for compounds acting against obesity.

Acute administration of serotonergic compounds alters the expression of peptidergic appetitive effectors within the hypothalamus, namely it causes an increase in anorectic pro-opiomelanocortin mRNA and a decrease in orexigenic neuropeptide Y mRNA (Choi et al. 2006). GABAergic neurons have an inhibitory effect on the activity of pro-opiomelanocortin neurons in the arcuate nucleus, which induces a hunger signal in the hypothalamus. It is believed that 5-HT_6_ receptor antagonists block the serotonin-dependent activation of GABA neurons, which results in reduction of inhibitory effects of GABA on pro-opiomelanocortin neurons in the arcuate nucleus with subsequent inhibition of the hunger signal induction (Sargent and Henderson 2011).

Various studies have demonstrated the influence of 5HT_6_ receptor ligands on body weight; it has been found that the anorexic effect of these compounds is associated with reduced food intake resulting from a mechanism that is consistent with an enhancement of satiety. On the other hand, the 5HT_6_ receptor ligands do not cause such clinically unacceptable effects as sedation, an emetic reflex, nausea and/or gastrointestinal malaise and conditioned taste aversion (Heal et al. 2008; Fisas et al. 2006). Additionally, the results from various experiments in animal models of human obesity predict that the weight-loss evoked by the 5-HT_6_ receptor ligands reduces cardio-metabolic risk profiles and causes an improvement of obesity-related metabolic diseases (Heal et al. 2008).

LuAE58054 – idalopirdine is a potent and selective 5-HT_6_ receptor antagonist (Arnt et al. 2010) under development by Lundbeck as an augmentation therapy for the treatment of cognitive deficits associated with Alzheimer’s disease and schizophrenia. Since February 2010 it has been in phase III clinical trials. LuAE58054 displays high affinity to the human 5-HT_6_ receptor with a Ki of 0.83 nm. In a 5-HT_6_ GTPgammaS efficacy assay, LuAE58054 showed no agonist activity, but demonstrated potent inhibition of 5-HT-mediated activation. Besides medium affinity to alpha_1A_- and alpha_1B_-adrenoreceptors, LuAE58054 demonstrated >50-fold selectivity for more than 70 targets examined (Arnt et al. 2010). Idalopirdine has previously been shown to be safe and well tolerated in doses of up to 360 mg (single dose) in clinical pharmacology studies (Wilkinson et al. 2014).

This study is the first to demonstrate, within the model of obesity, that during the treatment with LuAE58054 animals significantly lose weight, reaching the weight of animals without obesity, while being at the same time fed normal feed. Animals treated with LuAE58054 ate significantly less food when compared to the obese control animals. Reduced fat in the peritoneum and plasma triglycerides were also observed in this group of animals.

## Materials and methods

### Animals

The experiments were carried out on male Wistar rats; initial body weight was 140–160 g (24 animals in total). The animals were housed in pairs in plastic cages in constant temperature facilities exposed to 12–12 light–dark cycle. Water and food were available ad libitum. Control and experimental groups consisted of six to eight animals each. All experiments were conducted according to the guidelines of the Animal Use and Care Committee of the Jagiellonian University (2013, Poland).

### Experimental design

#### High-fat diet-induced obesity (DIO rats) and its influence on body weight, food and water intake

Sixteen male Wistar rats (150–160 g) were pair-housed and fed a fatty diet consisting of 40 % fat blend (Labofeed B with 40 % lard, Morawski, Manufacturer Feed, Poland) for 10 weeks with water available ad libitum. Control rats were fed a standard diet (Labofeed B, Morawski Manufacturer Feed, Poland) for 10 weeks. DIO-rats were randomly divided into two equal groups that had the same mean body weight and were treated intraperitoneally with a test compound (Lu AE58054) at 5 mg kg^−1^/day or a vehicle (5 % HP-beta-cyclodextrin) once per day in the morning between 9.00 and 10.00 for 21 days (Lu group or DIO-control group). Continually to the last day of the experiment, these animals consumed feed with 40 % fat. Intakes of food and water were measured three times per week, and body weight was measured daily, immediately prior to administration of drugs. On the twenty-first day, the food was discontinued and, in accordance with the standards, after a twenty-four hour period of fasting blood was collected for biochemical tests. On the 22st day, 20 min after intraperitoneal administration of heparin – 1000 units – to rats anesthetized with thiopental (70 mg kg^−1^ bw), blood was collected from the left common carotid artery. Plasma was obtained by centrifugation (4000 g/10 min) and then frozen at −80 °C. Peritoneal fat was collected from the rats and weighed.

### Determination of lipid profiles and glucose levels in plasma

To determine the lipid profile and glucose level in the plasma, standard enzymatic, spectrophotometric tests (Biomaxima S.A. Lublin, Poland) were used. The substrate was decomposed with appropriate enzymes for the relevant product, which was converted to a colored compound. Coloration was proportional to their concentration. The absorbance was measured at a wavelength of 500 (glucose, triglycerides, total cholesterol) or 600 nm (HDL, LDL).

### Locomotor activity test

The locomotor activity of rats was measured on the 20th day of the treatment with a special RFID-system – TraffiCage (TSE-Systems, Germany). The rats were housed in pairs in home cages with feed and water available ad libitum. The animals were subcutaneously implanted with transmitter identification (RFID), which enabled the presence and time spent in different areas of the cage to be counted and then the data was grouped in a special computer program.

### Data analysis and statistical procedures

Statistical calculations were carried out with the GraphPad Prism 6 program. The results were given as the arithmetic means with standard error of the mean (SEM). The statistical significance was calculated using the one-way ANOVA post-hoc Tukey Multiple Comparison Test or the two-way ANOVA post-hoc Bonferroni Multiple Comparison Test or the t-Student Test (for comparison two groups). Differences were considered statistically significant at: *p ≤ 0.05, **p ≤ 0.01, ***p ≤ 0.001.

### Drugs, chemical reagents and other materials

Heparin was delivered by Polfa Warszawa S.A. (Warsaw, Poland), while thiopental sodium was from Sandoz International (Stryków, Poland) and 5 % HP-beta-cyclodextrin from Sigma-Aldrich, USA.

Lu AE58054 was synthesized in the Chair of Pharmaceutical Chemistry, Faculty of Pharmacy, Jagiellonian University Medical College, Kraków, Poland according to a procedure described previously (Pasternak and Szymonifka 2009). The structure was confirmed by HNMR obtained in a Varian BB 200 spectrometer using TMS (0.00 ppm) in chloroform-d1. The purity was confirmed by UPLC/MS analysis to be > 98 %. The UPLC/MS system consisted of a Waters AQUITY UPLC® coupled to a Waters SQD mass spectrometer. Chromatographic separations were carried out using an Acquity UPLC® BEH C18 column, 2.1 × 100 mm and 1.7 μm particle size. The column was maintained at 60 °C, and eluted under gradient conditions: 4 min, a linear gradient from 80 to 0.1 % of eluent A at a flow rate of 0.5 ml/min. Eluent A: water/formic acid (0.02 %, *v*/*v*) and eluent B: methanol – acidic gradient. Eluent A: water/formic acid/ammonia solution (0.01 %/0.1 %, *v*/*v*/*v*) and Eluent B: methanol – alkaline gradient.

## Results

### Reduction of weight after LuAE58054 administration to DIO rats

**Fig 1:**
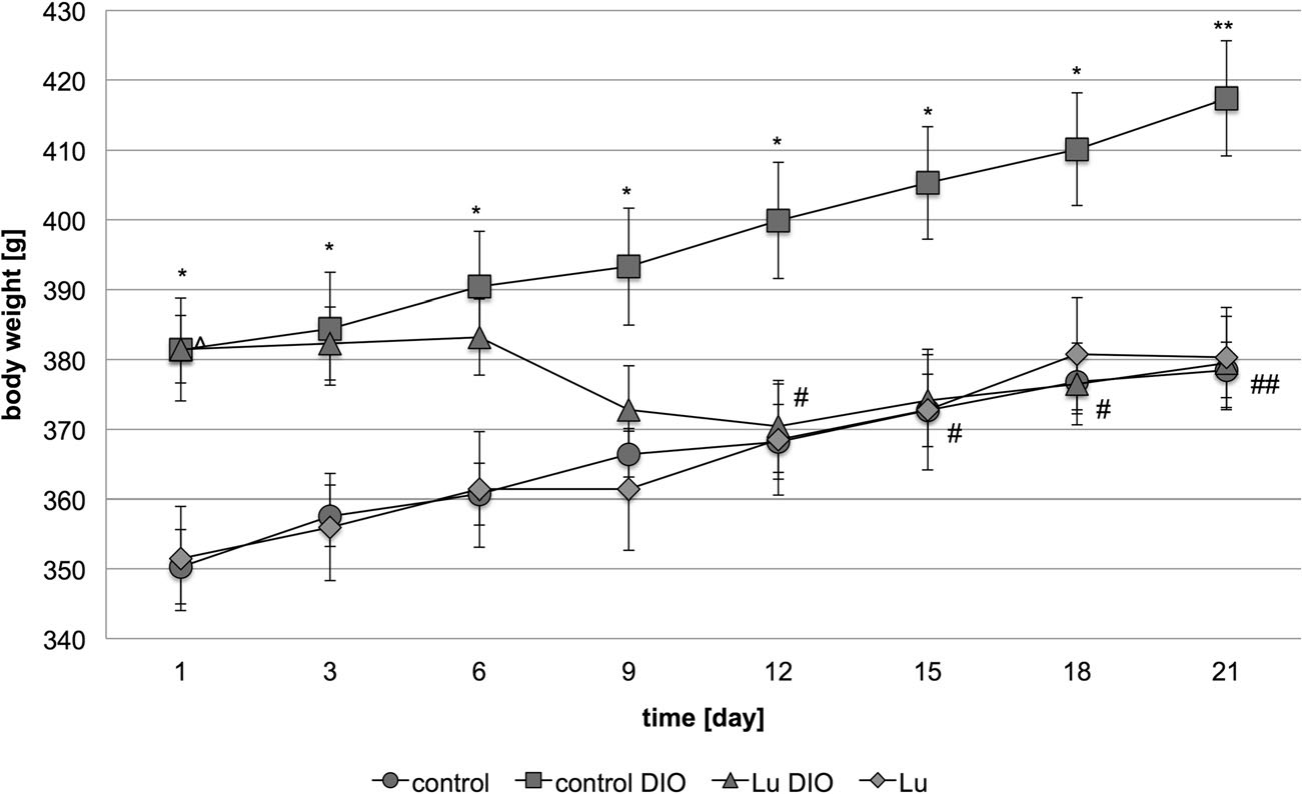
Effect of chronic administration of the 5-HT_6_ receptor antagonist, LuAE58054, on body weight in dietary-induced obese male Wistar rats. Results are means ± SEM, *n* = 6–8. Multiple comparisons of the vehicle-treated control group against the vehicle-treated DIO-control group (*) or LuAE58054-treated group against the vehicle-treated DIO-control group (#) or LuAE58054-treated group against the vehicle-treated control group (^) were by two-way ANOVA, post-hoc Benferroni test. Significant differences are denoted by *, #, ^ *p* < 0.05, **, ## *p* < 0.01

LuAE58054 administered intraperitoneally to rats fed on a high-fat feed caused a significant decrease in body weight gain compared to the obese rats (DIO-control group). It significantly reduced body weight to the level of the non-obese rats (control group - standard diet-fed rats) on the 12th day of treatment. Animals treated with LuAE58054 weighed 8 and 9.2 % less than the control obese animals on the 12th and 21st days, respectively. LuAE58054 did not influence body weight in rats fed a standard feed. The results are shown in Fig. 1.

### Influence of the test compound on the amount of peritoneal adipose tissue

**Fig 2:**
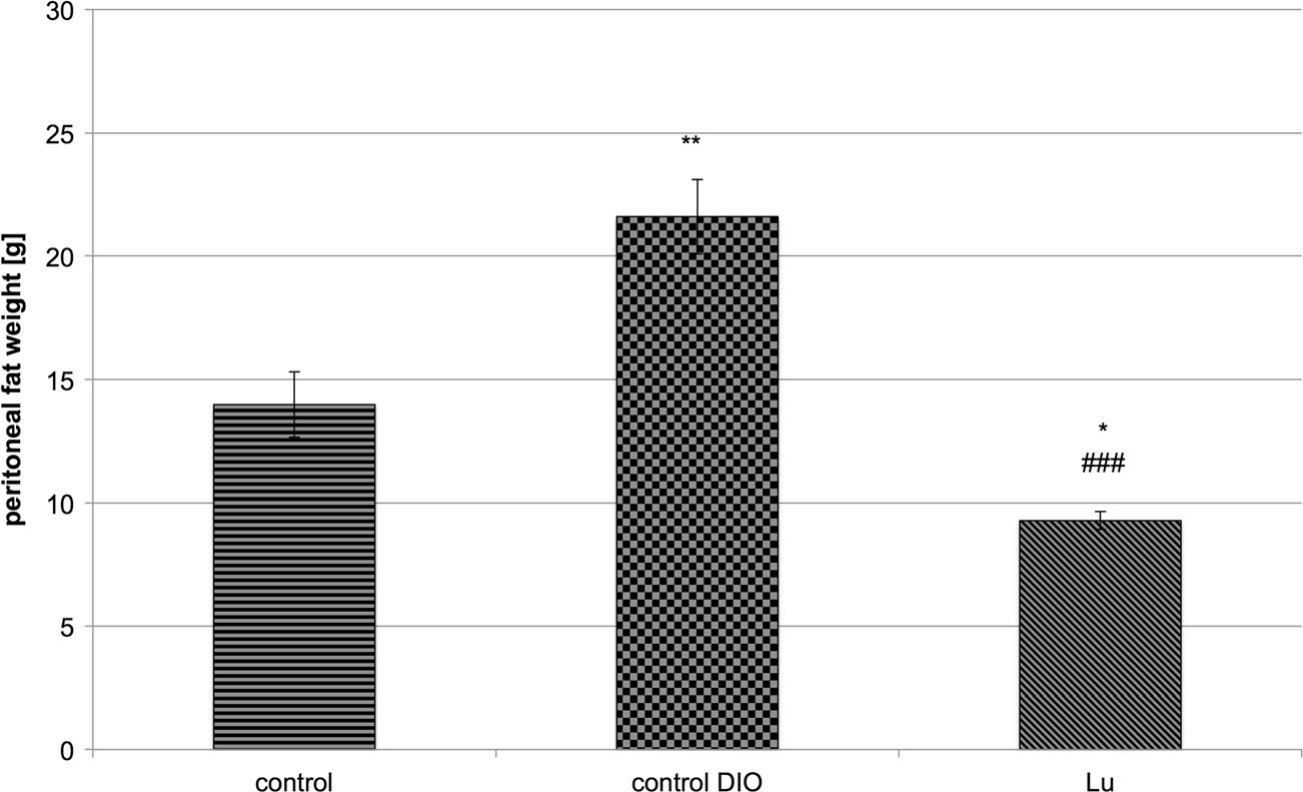
Weight of peritoneal fat after chronic administration of the 5-HT_6_ receptor antagonist – LuAE58054 – to male Wistar rats (control non-obese, control DIO obese, Lu – obese treated with LuAE58054). Results are means ± SEM, *n* = 6–8. Comparisons against the vehicle-treated control group (*) or against the DIO-control group (#) were by one-way ANOVA, post-hoc Tukey test. Significant differences are denoted by ** *p* < 0.01, ### *p* < 0.001

In the group receiving the test compound – LuAE58054 – the amount of fat in peritonea was significantly lower than in obese rats. Furthermore, the mass of adipose tissue was also significantly lower than in rats of the control, non-obese group, with a comparable weight of the whole body. The results are shown in Fig. 2.

### Influence of LuAE58054 on food and water intake

**Fig 3:**
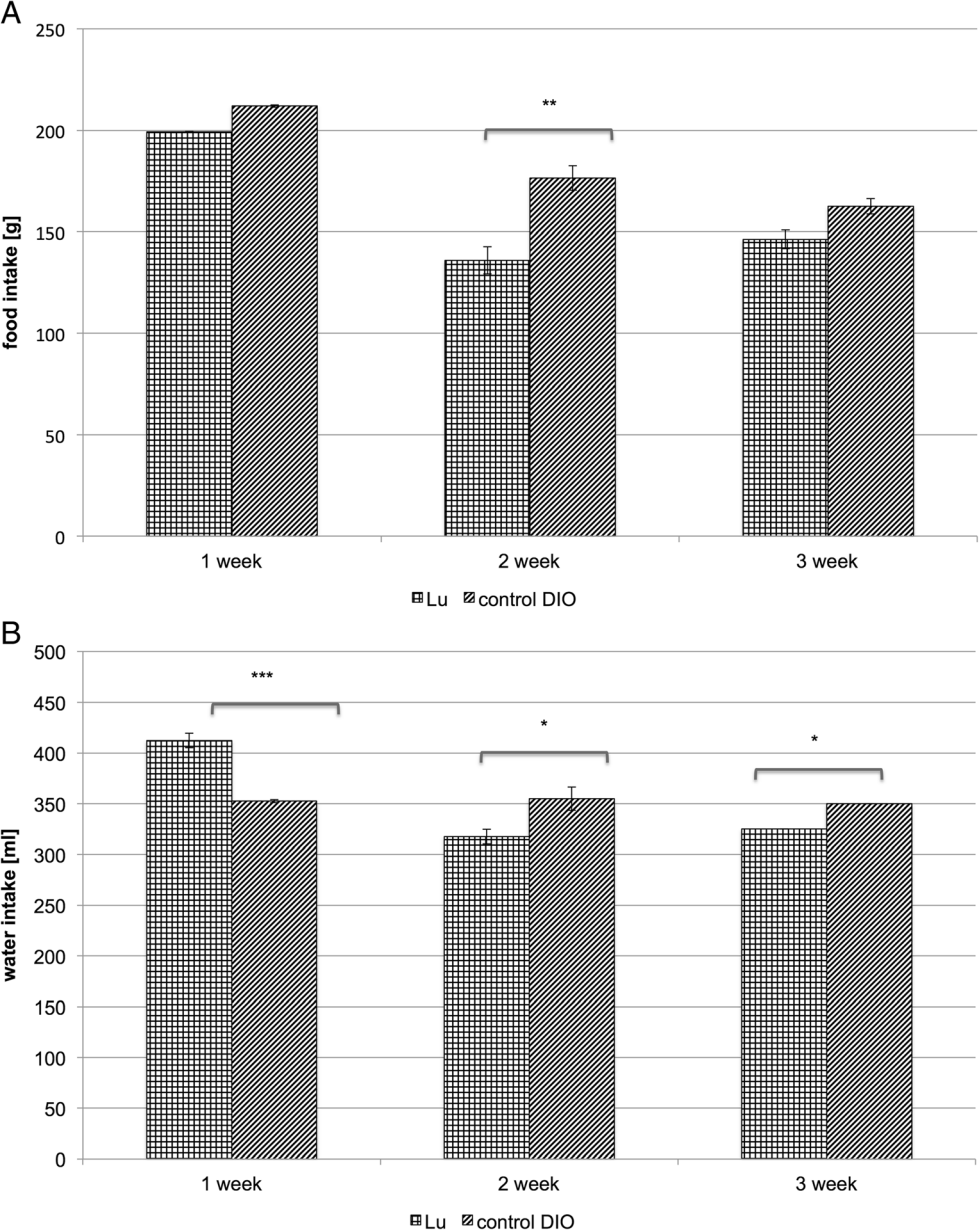
Effect of chronic administration of the 5-HT_6_ receptor antagonist, LuAE58054, on food intake (**a**) or water intake (**b**) in dietary-induced obese male Wistar rats. Results are means ± SEM, data for two animals reared together, *n* = 3–4. Multiple comparisons against the DIO-control group were by two-way ANOVA, post-hoc Bonferroni test. Significant differences are denoted by *p < 0.05, **p < 0.01, ***p < 0.001

There was a reduction of food intake in the group treated with LuAE58054 in comparison with the DIO-control group. Statistical significance was determined in the second week. In this period, weight loss was observed in treatment animals. The results are shown in Fig. 3a. In the first week of treatment with LuAE58054, a significant increase in water intake was observed in comparison with the intake in the DIO-control group, whereas in the next weeks of treatment a significant decrease in water consumption in rats treated with LuAE58054 was determined. The results are shown in Fig. 3b.

### Influence of chronic 21-day treatment with LuAE58054 on lipid and carbohydrate profiles in rats fed a high-fat diet

Treatment with Lu AE58054 for 21 days significantly decreased the level of triglycerides in plasma in rats fed with high-fat feed. This compound did not influence the blood glucose, total cholesterol and HDL cholesterol fraction or LDL. The results are shown in Table 1.

**Table 1:** Effects of chronic administration of the 5-HT_6_ receptor antagonist, LuAE58054, on glucose, triglycerides, total cholesterol, HDL cholesterol or LDL cholesterol levels in plasma control and dietary-induced obese male Wistar rats

	Glucose	TG	Cholesterol	HDL	LDL
Control	4.61	0.18	1.61	0.79	0.97
Control-DIO	5.70##	0.23#	3.62##	1.73##	1.83##
Idalopirdine	6.11##	0.17**	3.45##	1.77##	1.89##

Results are means ± SEM, *n* = 6–8. Concentrations in plasma: mmol/l. Comparisons against the DIO-control group were by t-Student test. Significant differences are denoted by **p < 0.01. Comparisons against the control group were by t-Student test. Significant differences are denoted by #*p* < 0.05 or ##*p* < 0.01

### Locomotor Activity after 20 days of treatment with Lu AE58054

**Fig 4:**
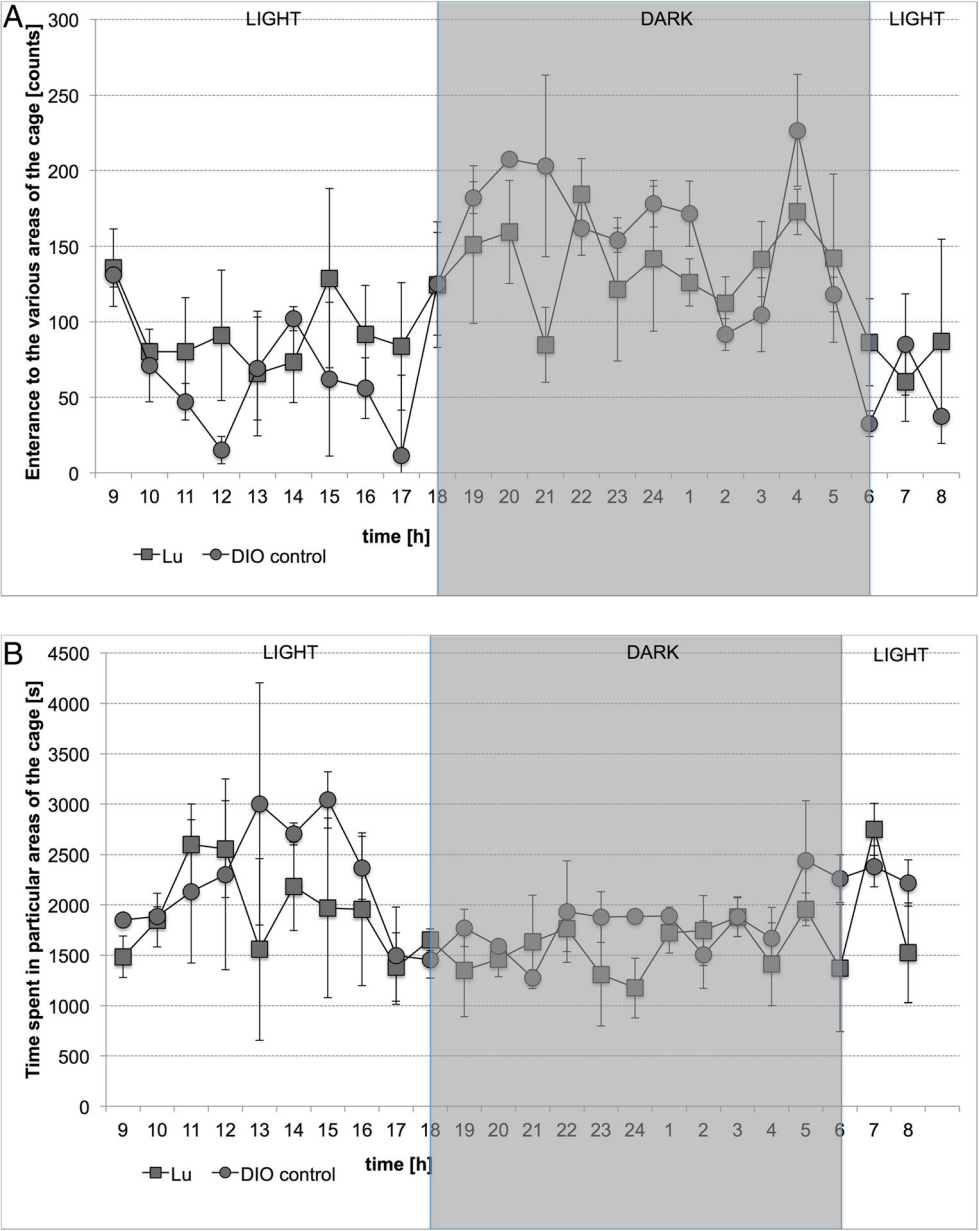
Locomotor Activity during 24 h after treatment with LuAE58054 or vehicle to DIO rats on 20th day of treatment. Activity is directly related to entering various areas of the cage (**a**) and inversely related to time spent in particular areas of the cage (**b**). Results are means, *n* = 6

In the group treated with the tested compound – Lu AE58054 – no statistically significant effect on the spontaneous activity of DIO rats was observed in comparison with the DIO-control group. Figure 4 shows the results.

## Discussion

Obesity leads to disruption of the functioning of the human body and an increase in the prevalence of various chronic diseases and mortality (Zięba 2007). Obese individuals are more likely to suffer from cardiovascular diseases (ischemic heart disease, hypertension, diabetes, and diseases of the digestive system) and they are at higher risk of developing colorectal, gallbladder, pancreas and kidney cancers. Treatment of obesity is very difficult and expensive. Development of additional diseases due to obesity also generates huge costs of treatment (World Obesity Federation 2014). Therefore, the search for compounds that effectively help reduce weight and show low risk for potential side effects is extremely important for the general public.

Oral administration to rats of LuAE58054 potently inhibited striatal in-vivo binding of the 5-HT_6_ antagonist radioligand [^3^H]LuAE60157, with an ED_50_ of 2.7 mg/kg. Steady-state modeling of an acute pharmacokinetic/5-HT_6_ receptor occupancy time-course experiment indicated a plasma EC_50_ value of 20 ng/ml. Administration of LuAE58054 in a dose range (5–20 mg/kg p.o.) led to more than 65 % striatal 5-HT_6_ receptor binding occupancy in vivo. These results indicate that LuAE58054 is a selective antagonist of 5-HT_6_ (Arnt et al. 2010).

Research into the influence of LuAE58054 (idalopirdine) on body weight in animals with diet-induced obesity was performed by intraperitoneal injection of 5 mg kg^−1^ bw of the compound for 21 days. This dose was the lowest effective dose in a rat model of cognitive impairment (Arnt et al. 2010). Intraperitoneal injection was chosen as an administration route to ensure that the high-fat diet did not influence the compound absorption. This route of administration has also been used in research into the influence on body weight of another 5-HT_6_ receptor antagonist - PRX-07034 - in a similar model of high-fat diet-induced obesity (Heal et al. 2008).

In safety and efficacy studies with idalopirdine in patients with moderate Alzheimer’s disease, this compound reduced body weight only in 5 % of patients, and this effect was not significant (Wilkinson et al. 2014). However, there was a notable difference between the elderly people who were the focus of the research and a population of obese people. Changes in body weight can affect the overall health of elderly patients and should not occur in these individuals. In particular, weight loss can lead to many health complications affecting daily activities, loss of functional status and mortality. Even looking for drugs that increase body weight in these people (Hilas and Avena-Woods 2014).

Our research has been conducted on the model of obesity. The research clearly showed a significant reduction of body fat and triglycerides in animals which were disrupted in these parameters – obesity model. This is the first report showing that idalopirdine has therapeutic potential against obesity.

Obesity is a major cause of insulin resistance, impaired glucose tolerance, type II diabetes, elevated plasma concentrations of triglycerides and low density cholesterol and decreased plasma concentrations of high density cholesterol and hypertension (Mokdad et al. 2001; Pi-Sunyer 2002). It is important, therefore, that in animals treated with LuAE58054 an anorectic effect occurred in parallel with lower plasma triglyceride levels compared to those in the obese control animals. After 21 days of treatment with Lu AE58054 the fat pads in peritoneal tissues of the rats were weighed and decreases in visceral fat depots were observed. Idalopirdine also caused a statistically significant reduction in visceral fat depots in rats without induced obesity. This is interesting, because there was no weight loss in rats fed standard feed treated with idalopirdine. Additional studies are, therefore, needed to determine why this occurred and what its cause was.

The present studies indicated that the reduction in food intake in obese animals during administration of LuAE58054 is associated with a reduction in their weight. This is consistent with the observations of many researchers who assert that the anorexic effect of 5-HT_6_ ligands is associated with reduced food intake via a mechanism that is consistent with an enhancement of satiety (Heal et al. 2008).

Administration of Lu resulted in statistically significant changes in water intake in different weeks of treatment. In the first week, a statistically significant increase in water consumption was observed compared to the intake of the control group. It is assumed that increased intake of water can reduce food intake (Muckelbauer et al. 2013). Those rats fed a high fat meal take less water than rats fed a standard feed (data not shown), which may be related to the fact that a high-fat feed in the form of a paste is not as dry as the standard feed. To understand why it is that in the second and third week a decline in the uptake of water is observed, it is necessary to look for the cause. In the literature there are no reports focusing on the effect of 5-HT_6_ ligands on water consumption, apart from the impact on water intake in a free-drinking test, where the SB-258585 – antagonist of these receptors did not affect the intake of water after a single administration (Wesołowska et al. 2007). It is known that the stimulation of 5HT_1A_ or 5-HT_1B_ receptors leads to a decrease in water consumption (Clissold et al. 2013). Increasing the water intake by animals can be associated with activation of metabolic processes, such as increases in both fatty acid oxidation and energy expenditure (Jbilo et al. 2005; Liu et al. 2005). The increase in drinking may be related to an increase in thermogenesis, as in studies with sibutramine administration in animals (Connoley et al. 1999; Skill et al. 2000). In the literature, there are other studies where sibutramina does not affect the level of water intake in rat models of diet-induced obesity (Pratt and Connolly 2010). Further detailed studies are needed to confirm the effect of idalopirdine and other serotonin receptor ligands on water consumption, and to ascertain whether this is linked with the effect of decreasing body weight.

Disorders of spontaneous activity (significant increase in activity as well as sedation) and the stress induced by the test compound could be very disadvantageous and could disturb an impact assessment of the test compound on body weight. This study demonstrated that repeated administration of Lu AE58054 to obese rats did not induce stress. It was essential to house animals in pairs in home cages. The test compound does not cause sedation. Therefore, the observed anorectic effect was definitely not associated with a reduction in the spontaneous activity of animals.

The anorectic effect of Lu AE58054 in the model of obesity where the animals were treated with high-fat feed ad libitum (DIO) was comparable to the anorectic effects of other 5-HT_6_ receptor ligands. In this model of obesity, 5 weeks of intraperitoneal treatment with PRX-07034 at the dose of 10 mg kg^−1^ bw/day caused a moderate decrease in rats’ food consumption and weight-loss (Heal et al. 2008). BVT 5182 – the other antagonist of the 5-HT_6_ receptor – was also shown to produce a reduction in food intake and body weight in DIO rats, simultaneous with decreases in visceral adiposity. This compound has presented anorectic activity in DIO mice and ob/ob mice too, when administered subcutaneously at a dose of 3 mg kg^−1^ bw (Heal et al. 2008). Ro 04–6790 at a high dose of 30 mg kg^−1^ i.p. also significantly attenuates body weight-gain in growing rats when given daily for 3 days (Woolley et al. 2001). E6837 – a partial agonist of the 5-HT_6_ receptor - when administered intragastrically at a dose of 30 mg kg^−1^ bw twice a day for 30 days reduces weight and food intake (Fisas et al. 2006). A comparison of the anorectic effects of Lu AE58054 and the mentioned ligands is presented in Table 2.

**Table 2:** Anorectic effect after chronic administration of certain compounds to DIO rats

	A	B	C	D	E	F
Compound	PRX-07034 10 mg/kg i.p. 35 days	E-6837 2* 30 mg/kg p.o. 28 days	silbutramine 3 mg/kg p.o. 21 days	silbutramine 5 mg/kg p.o. 28 days	silbutramine 7.5 mg/kg p.o. 28 days	Lu AE58054 5 mg/kg i.p. 21 days
Decrease of body weight	10.70 %	15.60 %	10 %	10.80 %	7.60 %	9.20 %

Percentage difference in comparison to DIO group at the end of treatment: A (Heal et al. 2008), B (Fisas et al. 2006), C (Brown et al. 2001), D. (Fisas et al. 2006), E. (Hansen et al. 2010), F. this study

The anorexic effect of Lu AE58054 shown in this study was also comparable to that of sibutramine which, when administered intragastrically for 21 days at a dose of 3 mg kg^−1^ bw/day to high-fat-fed obese rats, reduced their body weight by 10 % (Brown et al. 2001); while when administered at a dose of 5 mg kg^−1^ bw day for 28 days resulted in weight loss of 10.8 % (Fisas et al. 2006), and at a dose of 7.5 mg kg^−1^ bw/day for 28 days reduced body weight by 7.6 % (Hansen et al. 2010).

The proportion of patients achieving weight loss of at least 5 % ranges from 37 to 47 % for lorcaserin, 35 to 73 % for orlistat, and 67 to 70 % for top-dose phentermine plus topiramate–extended release. The safety and efficacy of these drugs have been determined for short-term use and there is limited data for their long-term use (Yanovski and Yanovski 2014). These data emphasize the need for drugs with different mechanisms of action, with improved efficacy and greater safety potential. 5-HT_6_ receptor antagonists do not cause disturbances in the cardiovascular system (Heal et al. 2008); therefore, the search for drugs against obesity in this group of drugs is particularly advantageous and important.

## Conclusion

This study is the first to use the model of obesity to demonstrate that animals lost a significant amount of weight during treatment with Lu AE58054. In dietary-induced obese rats, LuAE58054 led to significant signs of decreasing weight: reduced food intake, the amount of fat deposits in the peritoneum and the level of plasma triglycerides. Long-term administration did not lead to animal stress or sedation. The obtained data clearly indicate that idalopirdine is a compound with anti-obesity potential.
